# Comparison of 6 handheld ultrasound devices by point-of-care ultrasound experts: a cross-sectional study

**DOI:** 10.1186/s13089-024-00392-3

**Published:** 2024-10-02

**Authors:** Ariadna Perez-Sanchez, Gordon Johnson, Neysan Pucks, Riya N. Soni, Terry J. S. Lund, Anthony J. Andrade, Minh-Phuong T. Le, Jessica Solis-McCarthy, Tanping Wong, Arsal Ashraf, Andre D. Kumar, Gisela I. Banauch, James R. Verner, Amik Sodhi, Meghan K. Thomas, Charles LoPresti, Hannah Schmitz, Abhilash Koratala, John Hunninghake, Erik Manninen, Carolina Candotti, Taro Minami, Benji K. Mathews, Ghassan Bandak, Harald Sauthoff, Henry Mayo-Malasky, Joel Cho, Nick Villalobos, Kevin C. Proud, Brandon Boesch, Federico Fenton Portillo, Kreegan Reierson, Manpreet Malik, Firas Abbas, Tim Johnson, Elizabeth K. Haro, Michael J. Mader, Paul Mayo, Ricardo Franco-Sadud, Nilam J. Soni

**Affiliations:** 1 Division of Hospital Medicine, Joe R. Teresa Lozano Long School of Medicine, University of Texas Health San Antonio, 7703 Floyd Curl Drive, MC 7885, San Antonio, Texas 78229 USA; 2 Division of Hospital Medicine, Legacy Healthcare System, Portland, OR USA; 3 Section of Hospital Medicine, South Texas Veterans Health Care System, San Antonio, Texas USA; 4 Division of General Internal Medicine, Massachusetts General Hospital, Boston, MA USA; 5 Department of Emergency Medicine, Division of Ultrasound, Joe R. and Teresa Lozano Long School of Medicine, University of Texas Health San Antonio, San Antonio, Texas USA; 6 Division of Hospital Medicine, Weill Cornell Medicine, New York, NY USA; 7 Division of Hospital Medicine, Stanford University, Stanford, CA USA; 8 Division of Pulmonary & Critical Care Medicine, University of Massachusetts Chan Medical School, Worcester, MA USA; 9 Department of Hospital Medicine, HealthPartners Medical Group, Minneapolis-St. Paul, MN USA; 10 Division of Allergy, Pulmonary and Critical Care Medicine, University of Wisconsin School of Medicine and Public Health, Madison, WI USA; 11 Department of Medicine, Medical University of South Carolina, Charleston, South Carolina USA; 12 Department of Medicine, Case Western Reserve University School of Medicine, Cleveland, OH USA; 13 Department of Medicine, The Queen’s Medical Center, Honolulu, HI USA; 14 Division of Nephrology, Medical College of Wisconsin, Milwaukee, WI USA; 15 Department of Trauma, Brooke Army Medical Center, San Antonio, TX USA; 16 Division of Hospital Medicine, University of California Davis, Sacramento, CA USA; 17 Division of Pulmonary, Critical Care, and Sleep Medicine, The Warren Alpert Medical School of Brown University, Providence, Rhode Island USA; 18 Department of Internal Medicine, University of Minnesota, Minneapolis, MN USA; 19 Division of Nephrology, Joe R. and Teresa Lozano Long School of Medicine, University of Texas Health San Antonio, San Antonio, Texas USA; 20 Division of Pulmonary Diseases and Critical Care Medicine, Joe R. and Teresa Lozano Long School of Medicine, University of Texas Health San Antonio, San Antonio, Texas USA; 21 Division of Pulmonary, Critical Care, and Sleep Medicine, New York University Grossman School of Medicine, New York, NY USA; 22 Division of Pulmonary, Critical Care and Sleep Medicine, Westchester Medical Center, New York, USA; 23 Division of Pulmonary and Critical Care Medicine, NYC Health + Hospitals/Lincoln, New York, NY USA; 24 Department of Hospital Medicine, Kaiser Permanente Medical Center, San Francisco, CA USA; 25 Section of Pulmonary Medicine, South Texas Veterans Health Care System, San Antonio, Texas USA; 26 Cottage Medical Group, Cottage Health, Santa Barbara, CA USA; 27 Department of Internal Medicine, Washington State University, Elson S. Floyd College of Medicine, Everett, Washington USA; 28 Division of Hospital Medicine, Emory University School of Medicine, Atlanta, Georgia USA; 29 Department of Hospital Medicine, University of Arizona, Phoenix, AZ USA; 30 Division of Hospital Medicine, Virginia Commonwealth University Health, Richmond, VA USA; 31 Research and Development Service, South Texas Veterans Health Care System, San Antonio, Texas USA; 32 Department of Medicine, University of Central Florida, NCH Healthcare System, Naples, FL USA

**Keywords:** Point-of-care ultrasound, Handheld ultrasound, POCUS

## Abstract

**Background:**

Point-of-care ultrasound (POCUS) has emerged as an essential bedside tool for clinicians, but lack of access to ultrasound equipment has been a top barrier to POCUS use. Recently, several handheld ultrasound devices (“handhelds”) have become available, and clinicians are seeking data to guide purchasing decisions. Few comparative studies of different handhelds have been done. We conducted a cross-sectional study comparing 6 handhelds readily available in the United States (Butterfly iQ + ^™^ by Butterfly Network Inc.; Clarius^™^ by Clarius Mobile Health; Kosmos^™^ by EchoNous; TE Air^™^ by Mindray; Vscan Air^™^ SL and CL by General Electric; and Lumify^™^ by Philips Healthcare). A multi-specialty group of physician POCUS experts (n = 35) acquired three standard ultrasound views (abdominal right upper quadrant, cardiac apical 4-chamber, and superficial neck and lung views) in random order on the same standardized patients and rated the image quality. Afterward, a final survey of the overall ease of use, image quality, and satisfaction of each handheld was completed.

**Results:**

Thirty-five POCUS experts specializing in internal medicine/hospital medicine, critical care, emergency medicine, and nephrology acquired and rated right upper quadrant, apical 4-chamber, and superficial neck and lung views with 6 different handhelds. For image quality, the highest-rated handhelds were Vscan Air^™^ for the right upper quadrant view, Mindray TE Air^™^ for the cardiac apical 4-chamber view, and Lumify^™^ for superficial views of the neck and lung. Overall satisfaction with image quality was highest with Vscan Air^™^, Lumify^™^, and Mindray, while overall satisfaction with ease of use was highest with Vscan Air^™^. The 5 most desirable characteristics of handhelds were image quality, ease of use, portability, probe size, and battery life. Ultimately, all 6 handhelds had notable advantages and disadvantages, with no single device having all desired qualities or features.

**Conclusions:**

The overall satisfaction with image quality was rated highest with Vscan Air^™^, Lumify^™^, and Mindray TE Air^™^when acquiring right upper quadrant, apical 4-chamber, and superficial neck and lung views. No single handheld was perceived to be superior in image quality for all views. Vscan Air^™^ was rated highest for overall ease of use and was the most preferred handheld for purchase by POCUS experts.

**Supplementary Information:**

The online version contains supplementary material available at 10.1186/s13089-024-00392-3.

## Background

Point-of-care ultrasound (POCUS) is a powerful tool that has been shown to reduce procedural complications [1–4], improve bedside diagnostic accuracy [5], reduce diagnostic testing [6], and improve patient satisfaction [7, 8]. Despite the benefits, lack of access to an ultrasound machine has been a top barrier to POCUS use reported by multiple specialties [9–14]. Historically, the cost and size of cart-based ultrasound machines has limited their use in POCUS imaging. Since the 2010s, a surge of pocket-sized handheld ultrasound devices (“handhelds”) has dramatically improved clinicians’ access to portable ultrasound technology, especially in resource-limited settings [15, 16]. For the first time, handhelds have allowed clinicians to buy a personal ultrasound device for training and clinical use [17].

Although handhelds often have lower image quality, several studies comparing handhelds and cart-based ultrasound machines have demonstrated similar accuracy for common procedures and diagnoses, and any discrepant findings were not clinically significant [18–31]. However, few studies have compared different brands of handhelds in a head-to-head comparison [20, 32, 33]. One study compared 3 handhelds for gynecological ultrasound exams in a resource-limited setting [20], and another study evaluated 5 handhelds for ophthalmologic and facial aesthetics [32]. Based on our literature review, our group conducted the only head-to-head comparison of 4 handhelds for common general medical applications in December of 2021. Since then, major hardware and software updates have occurred to nearly all handhelds, and new handheld devices have become commercially available.

The objective of this study was to compare the performance of 6 common handheld ultrasound devices that are readily available in the United States to guide purchasing decisions. A multidisciplinary group of physician POCUS experts compared performance of handhelds to acquire 3 specific views (right upper quadrant, cardiac apical 4-chamber, and superficial neck and lung views) and rated the image quality. Afterward, experts rated the overall ease of use, image quality, and satisfaction of each device, and ranked the devices against each other. Additionally, we sought to identify the most important characteristics of handhelds per POCUS experts to guide selection of a device for use in clinical practice.

## Methods

### Subjects and setting

We conducted a cross-sectional study during a 2-day POCUS continuing medical education course in January of 2024. Thirty-five POCUS experts specializing in adult hospital medicine, critical care medicine, pulmonary medicine, emergency medicine, and nephrology acquired 3 standard POCUS views (right upper quadrant, apical 4-chamber, and superficial neck and lung views) using 6 commercially available handheld ultrasound devices on the same set of adult standardized patients with a body mass index (BMI) < 24. POCUS experts scanned the same patient with all devices for each of the 3 standard POCUS views. The University of Texas Health San Antonio Institutional Review Board reviewed and deemed this study to be non-regulated human research (STUDY00000326).

### Protocol

Six handheld ultrasound devices with both low- and high-frequency transducer capabilities were compared (Table 2): Butterfly iQ +^™^ (Butterfly Network, Inc.) all-in-one probe (referred to as “Butterfly iQ +^™^”) connected by a Lightning^®^ cable to an Apple iPad^®^ (iPad Pro^®^ 11-inch, iPad Air^®^ 11-inch); Clarius^™^ (Clarius Mobile Health) phased-array (PA HD3), linear (L15 HD3), and Convex (C3 HD3) probes (referred to as “Clarius^™^”) connected wirelessly to an Apple iPad^®^ (iPad Pro^®^ 11-inch); Kosmos^™^ (EchoNous, Inc.) linear (Lexsa) and phased-array (Torso-one) probes (referred to as “Kosmos^™^”) connected by a USB-C cable to an Apple iPad^®^ (iPad Pro^®^ 13-inch); TE Air^™^ (Mindray) phased-array probe (referred to as “Mindray”) connected wirelessly to an Apple iPad^®^ (iPad Pro^®^ 11-inch) and an Apple iPhone^®^ (iPhone 11 Pro^®^); Lumify^™^ (Philips Healthcare) probe (referred to as “Lumify^™^”) connected by a USB-C cable to a Samsung Galaxy S9 11-inch tablet^™^, and Vscan Air^™^ (GE Healthcare) SL (sector-phased array + linear) and CL (curved + linear) probes (referred to as “Vscan Air^™^”) connected wirelessly to a Samsung Galaxy A9 + 11-inch tablet^™^. Eight companies were requested to provide loaned handheld equipment only for this comparative study, but 3 companies (Exo, Vave Health, and Butterfly Network, Inc.) declined to provide equipment. Three Butterfly iQ +^™^ devices were provided by POCUS experts participating in this study; however, a sufficient number of handheld devices from Exo and Vave were not available for inclusion in the study.

Nine standardized patients were assigned to one of three POCUS views: (1) Focused Assessment with Sonography in Trauma (FAST) right upper quadrant (RUQ) view (diaphragm, liver, hepatorenal recess, and right kidney), (2) apical 4-chamber and 5-chamber views of the heart, (3) superficial view of the right neck (thyroid, internal jugular vein, and common carotid artery) and lung along the anterior chest wall (ribs, pleural line with lung sliding). Standardized patients were pre-scanned by 2 POCUS experts with a cart-based machine (Sonosite PX^™^ Fujifilm-Sonosite) and selected if high-quality images of one of the 3 views could be easily obtained based on their expertise.

Using the 6 handheld devices, all 35 POCUS experts independently acquired the same views on the same standardized patients. For the RUQ view, experts were instructed to use the curvilinear transducer, except for Mindray and Kosmos^™^ which only had phased-array transducers and Butterfly^™^ which had an all-in-one transducer. All RUQ views were acquired with an abdominal preset and focused on the liver, kidney, diaphragm, aorta, and spine. Color flow Doppler was applied over the vessels in the renal pelvis. For the apical 4-chamber view, experts were instructed to use the phased-array transducer with a cardiac preset to acquire views of the mitral valve, aortic valve, and right and left atria and ventricles. Experts were instructed to focus on the resolution of the endocardial lining and cardiac motion. Color flow Doppler was then applied over the mitral valve and left ventricular outflow tract. For the transverse view of the neck and superficial view of the lung, experts were instructed to use the high-frequency linear transducer with a venous or vascular preset to acquire transverse views of the internal jugular vein, common carotid artery, and thyroid gland, and color flow Doppler was applied over the common carotid artery and internal jugular vein. Next, a lung preset was used to acquire longitudinal views of the lung on the anterior chest wall to visualize lung sliding. All handhelds, except Mindray, had a high-frequency linear transducer or lung preset.

### Data collection

**Fig. 1:**
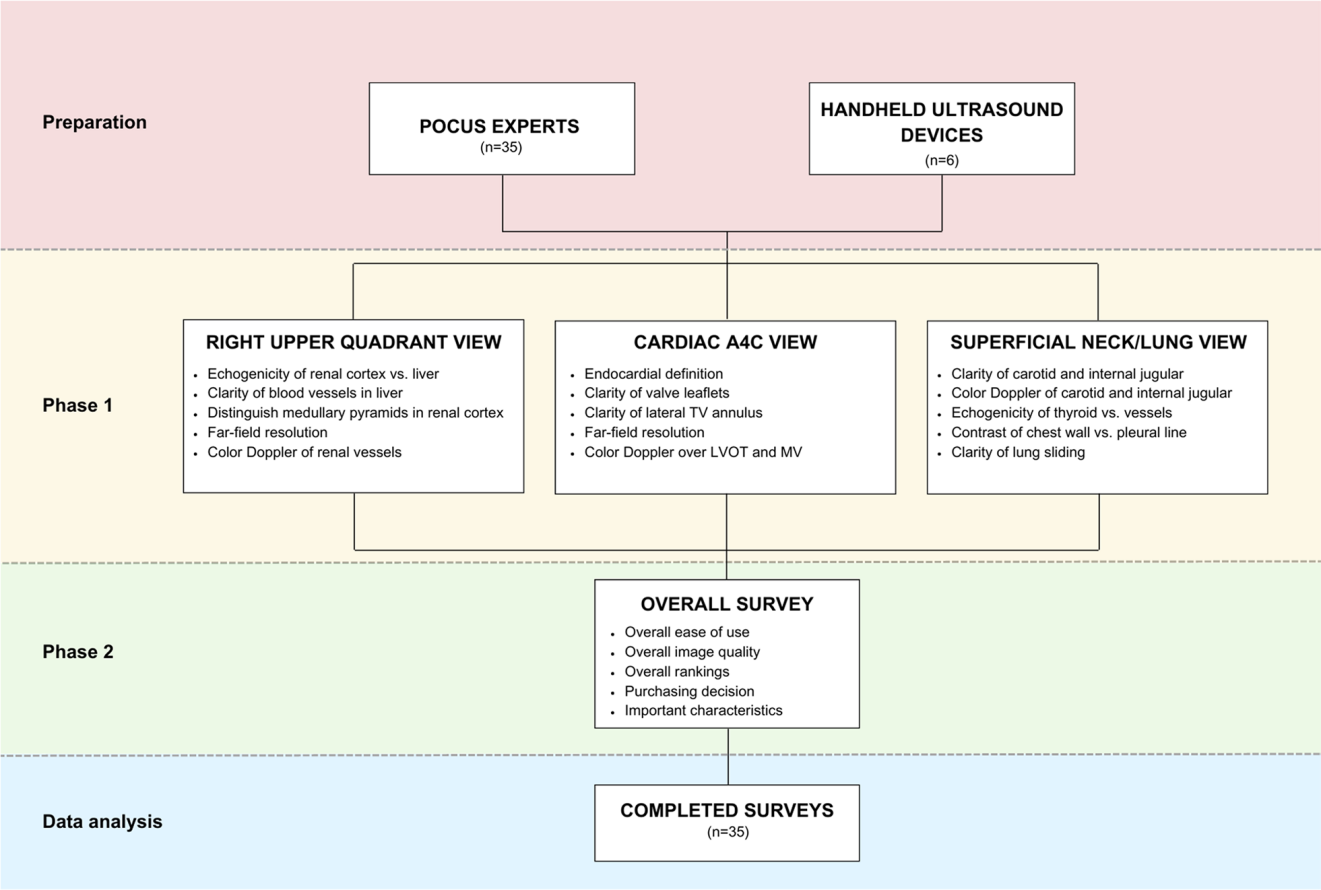
Study Flow Diagram. POCUS, point-of-care ultrasound. *A4C* apical 4-chamber, *TV* tricuspid valve, *LVOT* left ventricular outflow tract, *MV* mitral valve

This study was conducted in two phases (Fig. 1). First, experts rated the image quality of the 6 handheld devices for each of the 3 views as 0 (“poor”), 1 (“interpretable”), 2 (“good”), or 3 (“excellent”). Specific anchors were provided to rate the image quality for 5 characteristics of each view on the data collection forms (Additional files 1–3). An overall ranking of each device from 1 (“best”) to 6 (“worst”) was performed for each view. Second, data were collected on the overall ease of use, image quality, and satisfaction of each device (“overall survey”) (Additional File 4). For ease of use, experts rated the physical characteristics, software navigation, maneuverability of the probe/tablet for imaging, and overall satisfaction. For image quality, experts rated the detail resolution, contrast resolution, penetration, clutter, and overall satisfaction. The overall ranking assessed satisfaction and recommendation for purchase. Ratings were made using standardized statements on a Likert scale of 1 (”strongly disagree” or “very dissatisfied”) to 5 (“strongly agree” or “very satisfied”). Qualitative feedback was collected in each category using free text. Experts completed all data collection forms immediately and the overall survey no later than 72 h after scanning each standardized patient. Data were captured electronically using REDCap^™^ (Vanderbilt University, Nashville, TN, USA).

### Data analysis

Descriptive statistics about the experts were reported as frequencies with percentages, without any statistical analysis. Ratings of ease of use and image quality were compared using the Kruskal–Wallis rank sum test, with the Dwass-Steel-Critchlow-Fligner post hoc method to control the familywise-error rate. Rank analysis was performed via Friedman’s test, followed by a post hoc Sign test for paired data, using the Holm’s step-down procedure to control the familywise-error rate. For image quality ratings of the 3 specific views, scores were calculated by finding the mean score of each characteristic across raters and then adding the 5 means within a view, while the comparison of devices was done using a non-linear mixed model to predict the rating scores, with the device and view characteristic as fixed factors and rater as a random factor.

Potential bias due to prior experience with a handheld was assessed by having experts rate their past experience with a device as none (1), some (2), or proficient (3). Spearman correlation coefficients were calculated to evaluate the correlation between experts’ prior experience with using each handheld device and ratings for ease-of-use, image quality, and overall satisfaction, with a modified independent sample t-test used to test for statistical significance. A p-value < 0.05 denoted statistical significance. All analyses were performed with SAS software version 9.4.

Free text responses were analyzed using a qualitative deductive and inductive coding process based on a framework method approach. Advantages and disadvantages of all 6 handhelds were coded and tabulated. Two investigators independently applied the coding framework to the free text responses, resolved coding differences through discussion, and assigned a final code based on that discussion.

## Results

### POCUS experts

Thirty-five POCUS experts specializing in internal medicine/hospital medicine, critical care, emergency medicine, and nephrology that care for adult patients participated in this study. Most experts (80%) had either completed a POCUS training certificate through a national specialty society, achieved certification through the National Board of Echocardiography, or completed a dedicated POCUS fellowship, and 75% had > 5 years of experience using POCUS to guide patient care (Table 1). Right upper quadrant, apical 4-chamber, and superficial neck and lung views were acquired and rated by each of the POCUS experts using 6 different handhelds on the same adult standardized patients.

**Table 1:** Characteristics of the point-of-care ultrasound experts

Characteristic	All Experts (%) n = 35
**Specialty**	
Hospital medicine	22 (64)
Pulmonary and critical care medicine	8 (22)
Critical care medicine	3 (8)
Emergency medicine	1 (3)
Nephrology	1 ( 3)
**Gender**	
Female	9 (26)
Male	26 (74)
**United States Region**	
South (TX, FL, GA, VA, SC)	14 (40)
Northeast (NY, MA, RI)	7 (20)
West (CA, OR, AZ, HI)	8 (22)
Midwest (MN, WI, OH)	6 (17)
**Past ultrasound training**	
Certificate program^1^	14 (40)
National board of echocardiography^2^	14 (40)
Ultrasound fellowship	2 (5)
**Clinical experience in practice**	
0–5 years	10 (28)
6–10 years	10 (31)
> 10 years	15 (42)
**Experience using point-of-care ultrasound**	
0–5 years	9 (25)
6–10 years	14 (42)
> 10 years	12 (33)
**Applications routinely used**^3^	
Procedural guidance	29 ( 83)
Cardiac	35 (100)
Pulmonary	35 (100)
Abdomen	33 (94)
Vascular	28 (80)
Skin/soft tissues	24 (69)

^1^ Training certificates offered by the Certificate of Completion program by the American College of Chest Physicians (CHEST) or the Society of Hospital Medicine

^2^ Either testamur status or full certification in Advanced Critical Care Echocardiography or Certification for Adult Echocardiography

^3^ Experts were allowed to select more than one application and each application represents a percentage of 35 experts

### Handheld characteristics

Characteristics of the 6 handhelds compared in this study are shown in Table 2. All handhelds had M-mode and color flow Doppler imaging modes, but only Kosmos^™^ had continuous-wave Doppler. All handhelds, except Mindray, were compatible with both iOS and Android tablets. Clarius^™^, Mindray, and Vscan Air^™^ were wireless. Butterfly iQ + ^™^ and Vscan Air^™^ were multifunctional transducers allowing acquisition of cardiac, abdominal, and superficial images from the same transducer, while Mindray allowed acquisition of both cardiac and abdominal images.

**Table 2:** Characteristics of Handheld Ultrasound Devices

	Modes^1^						Probe types & characteristics						Study views			APPROXIMATE COST (per probe)^2^
	MM	CFD	PD	TDI	PWD	CWD	All-in-one	Size	Weight	Wired	Wireless	iOS vs. Android	Abdomen RUQ view	Cardiac A4C view	Superficial Neck/Lung view	
**Butterfly iQ+**^**TM**^																
Butterfly iQ +	✔	✔	✔		✔		✔	56 × 35 × 163 mm	309 g	✔		iOS + Android	✔	✔	✔	$3,500 or $2,700 + $420/yr
**Clarius**																
Phased Array (PA HD3)	✔	✔	✔		✔			148 × 76 × 32 mm	292 g		✔	iOS + Android		✔		$3,600–$5,400 + $595/yr
Linear (L15 HD3)	✔	✔	✔		✔			147 × 76 × 32 mm	290 g		✔				✔	
Convex (C3 HD3)	✔	✔	✔		✔			146 × 76 × 32 mm	308 g		✔		✔			
**Kosmos**																
Linear (Lexsa)	✔	✔	✔		✔			155 × 56 × 35 mm	280 g	✔		iOS + Android			✔	$4,500
Phased-array (Torso-one)	✔	✔	✔	✔	✔	✔		150 × 56 × 35 mm	275 g	✔			✔	✔		
**Lumify**																
Sector (S4-1)	✔	✔			✔			102 × 55 mm	96 g	✔		iOS + Android		✔		$5,250
Linear (L12-4)	✔	✔			✔			114 × 45 mm	108 g	✔					✔	
Curved (C5-2)	✔	✔			✔			114 × 45 mm	136 g	✔			✔			
**Mindray**																
Mindray TE Air	✔	✔	✔	✔	✔		✔	33 × 47 × 170 mm	198 g		✔	iOS	✔	✔		$6,000–$8,000
**Vscan air**																
Sector-phased array + Linear (SL)	✔	✔			✔			141 × 67 × 33 mm	218 g		✔	iOS + Android		✔	✔	$4,500
Curved + Linear (CL)	✔	✔			✔			131 × 64 × 31 mm	205 g		✔		✔		✔	

^1^Imaging modes in addition to 2-dimensional or B-mode

^2^Approximate cost per probe in January–February 2024 which does not include the cost of a tablet

*MM* M-mode, *CFD* color-flow Doppler, *PD* Power Doppler, *TDI* tissue Doppler imaging, *PWD* pulsed-wave Doppler, *CWD* continuous wave Doppler, *RUQ* right upper quadrant, *A4C* apical 4-chamber

### Specific views

#### Abdominal right upper quadrant view

**Fig. 2:**
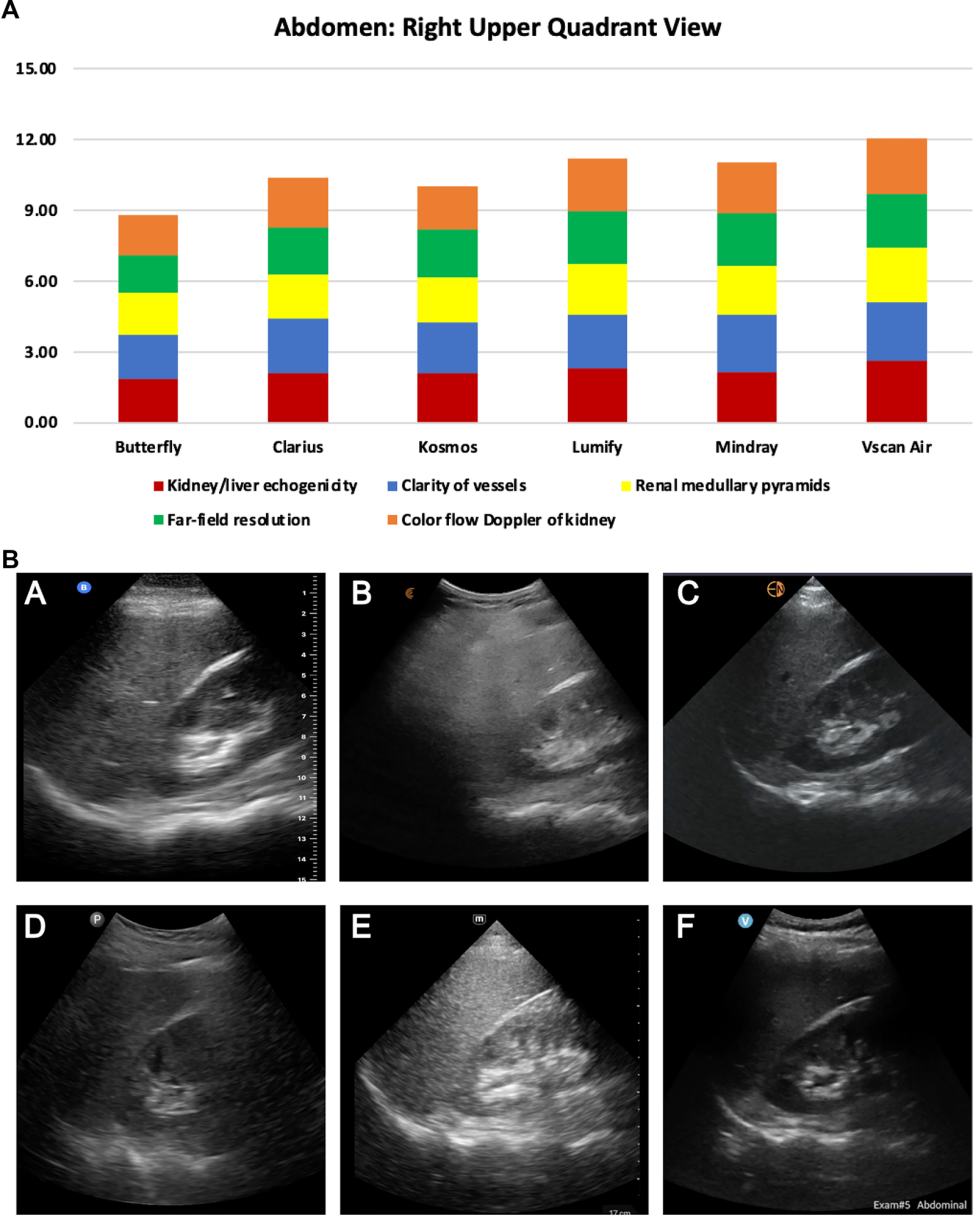
**A)** Abdominal Right Upper Quadrant View ratings of image quality by handheld (5 domains displayed were rated on a scale from 0 to 3); **B)** Abdominal Right Upper Quadrant View acquired from the same standardized patient showing kidney, liver, and diaphragm from 6 handheld devices: **A** Butterfly iQ +^™^, **B** Clarius^™^, **C** Kosmos^™^, **D** Lumify^™^, **E** Mindray, and **F** Vscan Air^™^

The specific characteristics evaluated in the RUQ view were the difference in echogenicity of the renal cortex and liver, clarity of blood vessels in the liver parenchyma, distinction of the medullary pyramids in the renal cortex, far-field resolution, and color flow Doppler of vessels in the renal pelvis. For the abdominal RUQ view, the top 3 highest-rated handhelds were Vscan Air^™^, Lumify^™^, and Mindray (Fig. 2) which was consistent with the overall ranking for the RUQ view (Additional File 5: Table S1).

#### Cardiac apical 4-chamber view

**Fig. 3:**
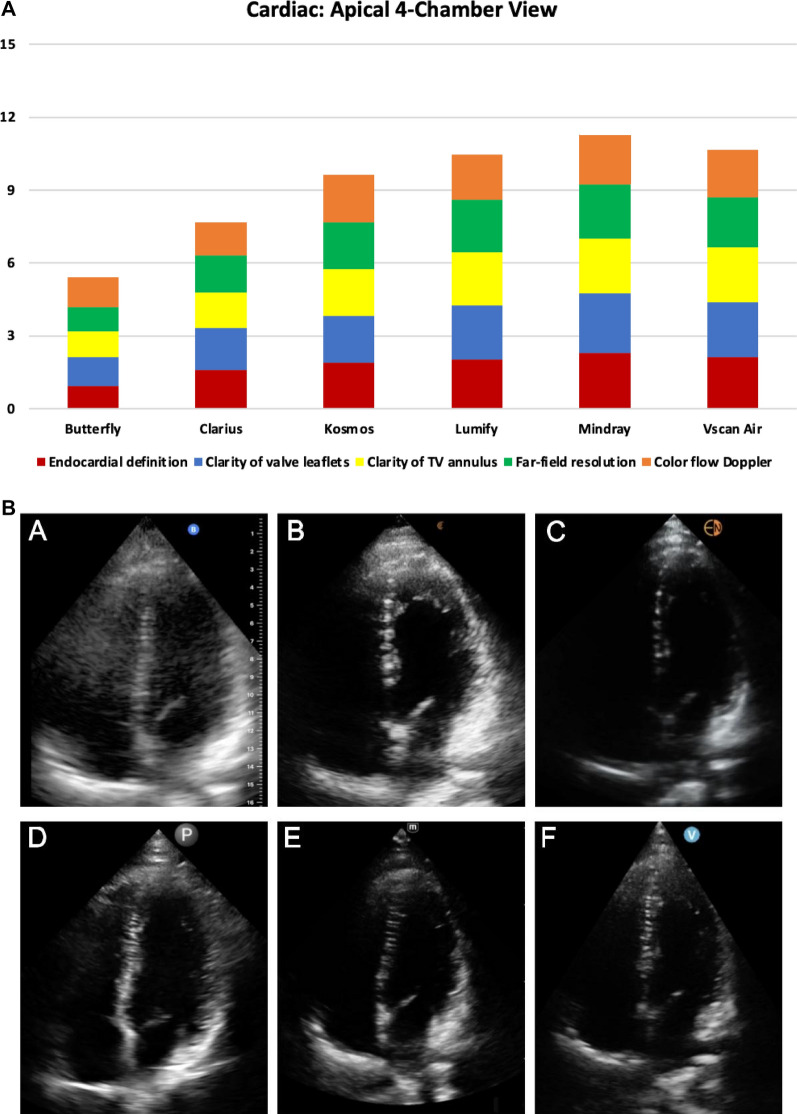

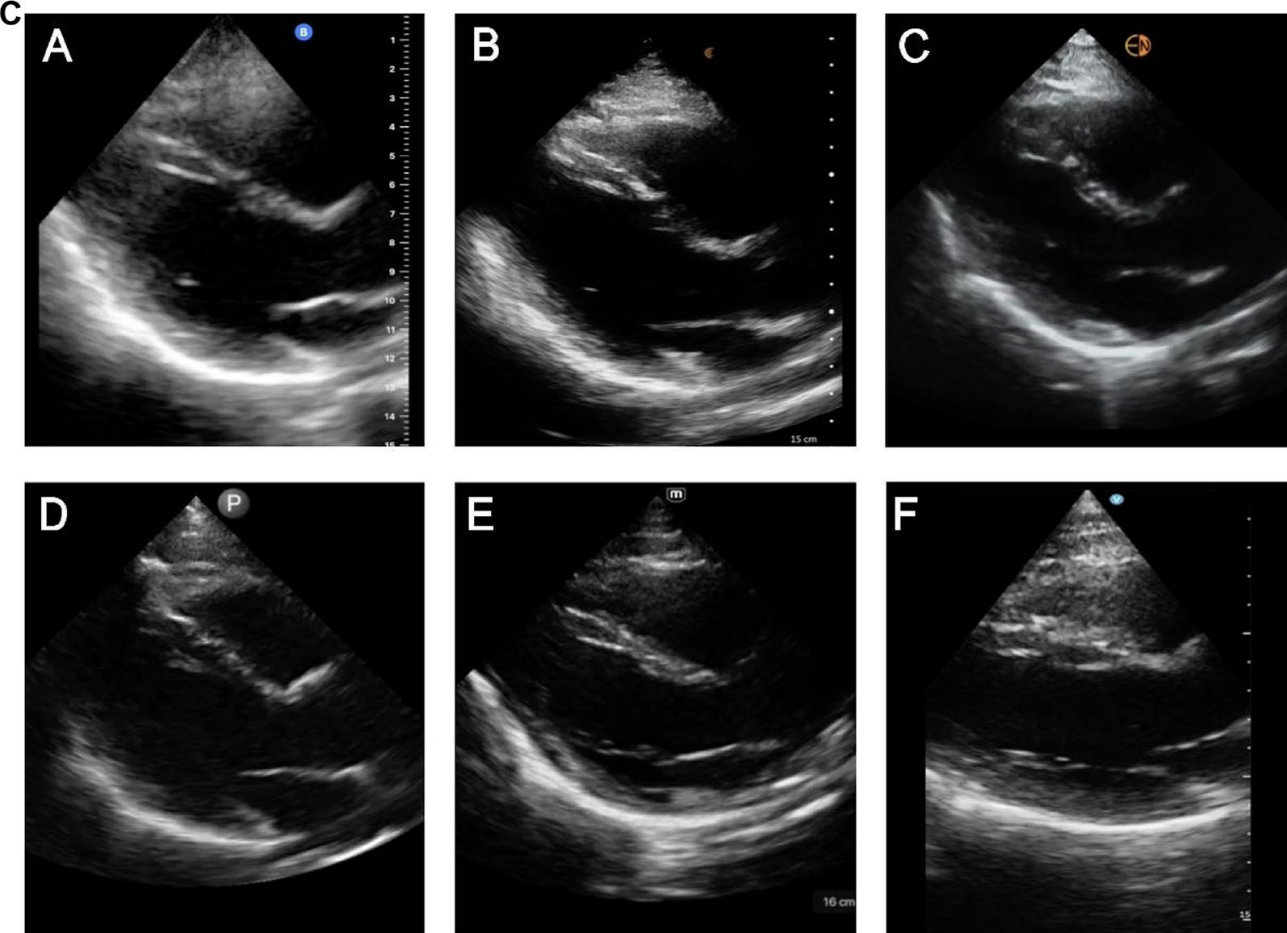
**A) **Cardiac Apical 4-chamber View ratings of image quality by handheld (5 domains displayed were rated on a scale from 0 to 3); **B)** Cardiac Apical 4-chamber View acquired from the same standardized patient in mid-diastole with the mitral and tricuspid valves open from 6 handheld devices: **A** Butterfly iQ +^™^, **B** Clarius^™^, **C** Kosmos^™^, **D** Lumify^™^, **E** Mindray, and **F** Vscan Air^™^; **C** Cardiac Parasternal Long-axis View acquired from the same standardized patient in early systole with the mitral valve closed and aortic valve open from 6 handheld devices: **A** Butterfly iQ + ^™^, **B** Clarius^™^, **C** Kosmos^™^, **D** Lumify^™^, **E** Mindray, and **F** Vscan Air^™^

The specific characteristics evaluated in the apical 4-chamber view were endocardial definition, clarity of valve leaflets, clarity of the lateral tricuspid valve annulus, far-field resolution, and color flow Doppler of the left ventricular outflow tract and mitral valve. For the apical 4-chamber view, the top 3 highest-rated handhelds were Mindray, Vscan Air^™^, and Lumify^™^ which was consistent with the overall ranking (Fig. 3 and Additional File 5: Table S2). Compared to the RUQ view and superficial neck and lung views, the total rating scores for the apical 4-chamber view were lower with all handhelds. Parasternal long-axis views were not rated in this study, but sample images acquired from a standardized patient post-study are provided for the benefit of readers (Fig. 3C).

#### Superficial neck and lung views

**Fig. 4:**
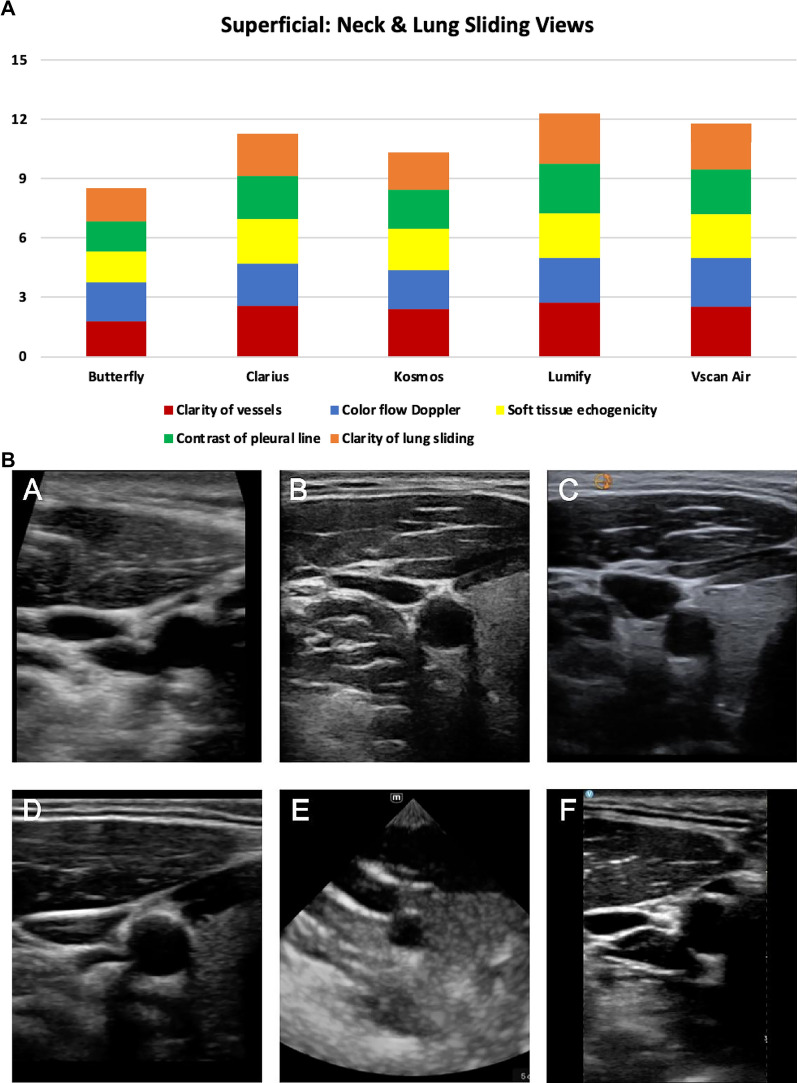

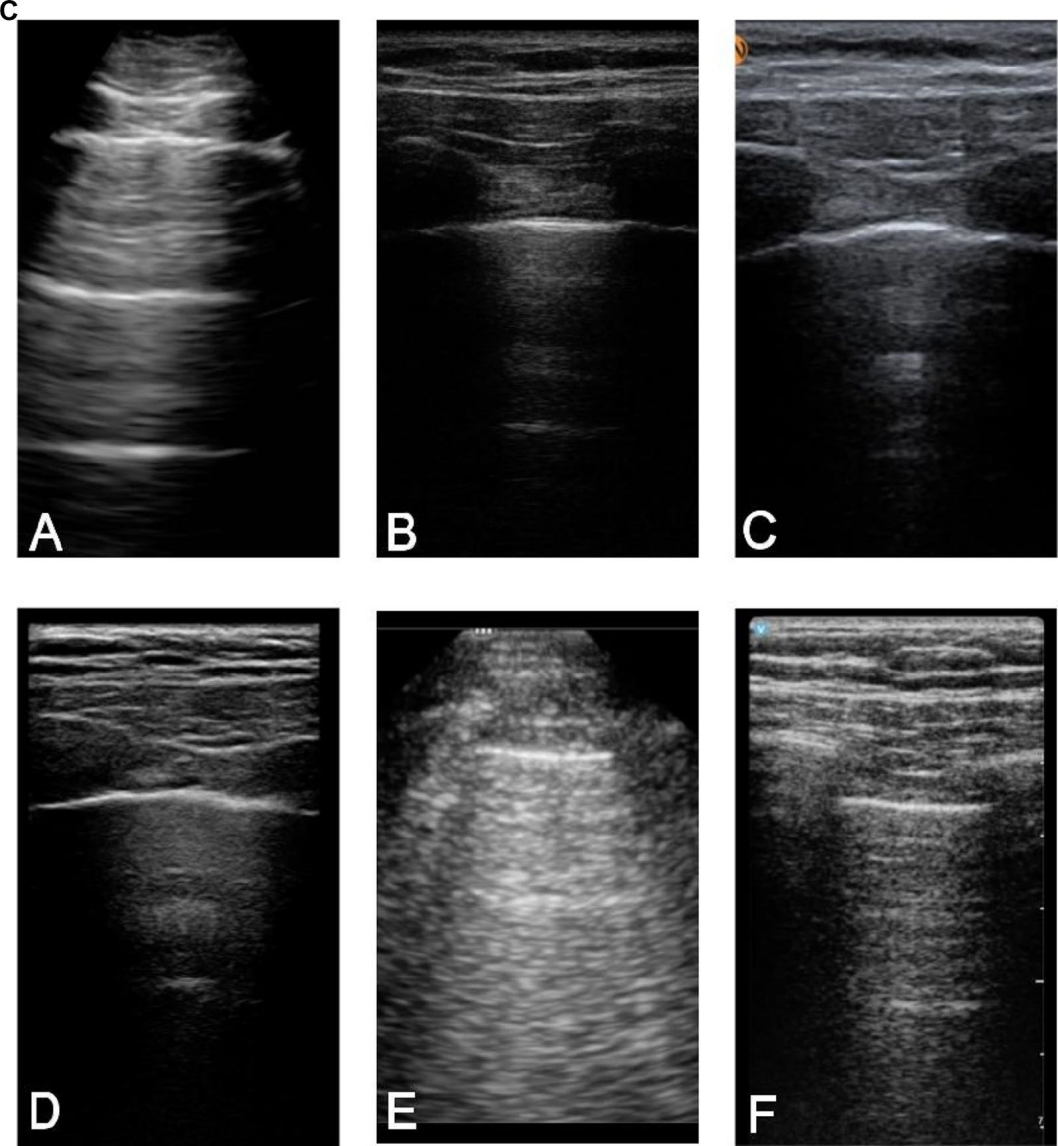
**A)** Superficial Neck and Lung Sliding View ratings of image quality by handheld (5 domains displayed were rated on a scale from 0 to 3); **B)** Superficial Neck Views acquired from the same standardized patient displaying the thyroid, common carotid artery, and internal jugular vein from 6 handheld devices: **A** Butterfly iQ +^™^, **B** Clarius^™^, **C** Kosmos^™^, **D** Lumify^™^, **E** Mindray, and **F** Vscan Air^™^; **C)** Superficial Lung Views acquired from the same standardized patient showing the pleural line from 6 handheld devices: **A** Butterfly iQ +^™^, **B** Clarius^™^, **C** Kosmos^™^, **D** Lumify^™^, **E** Mindray, and **F** Vscan Air^™^. Mindray lacks a linear probe and was excluded from the comparison of superficial views of the neck and lungs. The Mindray images in sections **B** (panel **E**) and **C** (panel **E**) are only displayed for demonstration purposes

The specific characteristics evaluated in the superficial neck and lung views were clarity of the carotid artery/internal jugular vein, color flow Doppler of carotid artery/internal jugular vein, difference in echogenicity of thyroid, contrast of chest wall vs. pleural line, and clarity of lung sliding. For the superficial views, the top 3 highest-rated handhelds were Lumify^™^, Vscan Air^™^, and Clarius^™^ which was consistent with the overall ranking (Fig. 4); however, the difference in image quality between the Vscan Air™ and Lumify™ was not statistically significant (Additional File 5: Table S3). Notably, the Mindray handheld lacked a linear probe and was excluded from the comparison of superficial views.

### Overall survey

After rating the specific views, all 35 POCUS experts completed an overall survey on ease of use, image quality, and satisfaction of each device. Specific characteristics and ratings for ease of use and image quality are shown in Table 3. Vscan Air^™^ and Mindray were rated the highest on physical probe characteristics and maneuverability, while Vscan Air^™^ and Butterfly iQ +^™^ were rated highest for ease of use of their software. For overall satisfaction with ease of use, Vscan Air^™^ was rated highest followed by Lumify^™^ and Mindray.

**Table 3:** Overall ease of use & image quality ratings of handheld ultrasound devices per experts (n = 35)

Variable [Mean score (s.d.)]	Butterfly iQ + ^TM^	Clarius	Kosmos	Lumify	Mindray	Vscan Air	p-value
**Ease of use**^**TM**^							
Physical characteristics	3.17 (1.1)	2.66 (1.2)	3.31 (1.1)	***4.31 (0.6)***	***4.49 (0.7)***	***4.49 (0.8)***	< 0.0001
Software	***4.23 (0.8)***	3.49 (0.7)	***3.89 (1.1)***	***3.86 (0.9)***	3.49 (1.1)	***4.34 (0.9)***	< 0.0001
Maneuverability	3.80 (1.0)	3.20 (1.1)	3.54 (1.0)	3.83 (0.9)	***4.23 (0.8)***	***4.43 (0.9)***	< 0.0001
Overall satisfaction	3.34 (1.0)	2.91 (1.1)	3.66 (1.0)	4.11 (0.9)	4.06 (0.9)	***4.63 (0.6)***	< 0.0001
**Image quality**^**TM**^							
Detail resolution	2.83 (1.0)	3.86 (0.8)	***4.11 (0.8)***	***4.26 (0.7)***	***4.17 (1.0)***	***4.54 (0.6)***	< 0.0001
Contrast resolution	2.69 (0.9)	3.83 (0.7)	4.03 (0.7)	***4.17 (0.7)***	***4.26 (0.8)***	***4.57 (0.6)***	< 0.0001
Penetration	2.71 (1.0)	3.63 (0.6)	***3.97 (0.9)***	***4.06 (0.9)***	***4.17 (0.9)***	***4.43 (0.7)***	< 0.0001
Clutter	2.34 (0.9)	3.63 (0.8)	***3.86 (0.9)***	***3.86 (0.8)***	***4.11 (0.9)***	***4.40 (0.6)***	< 0.0001
Overall satisfaction	2.37 (0.9)	3.69 (0.8)	3.74 (1.0)	***4.20 (0.9)***	***4.11 (0.9)***	***4.57 (0.6)***	< 0.0001

5 = Strongly agree; 4 = Agree; 3 = Neutral; 2 = Disagree; 1 = Strongly disagree

p-values from Kruskal–Wallis rank sum test; < 0.05 indicates at least one device is statistically different from another device

The highest scoring device in each row, and any devices that do not have a statistically significant difference in score using the Dwass-Steel-Critchlow-Fligner post hoc method, are presented in bold italic

**Fig. 5:**
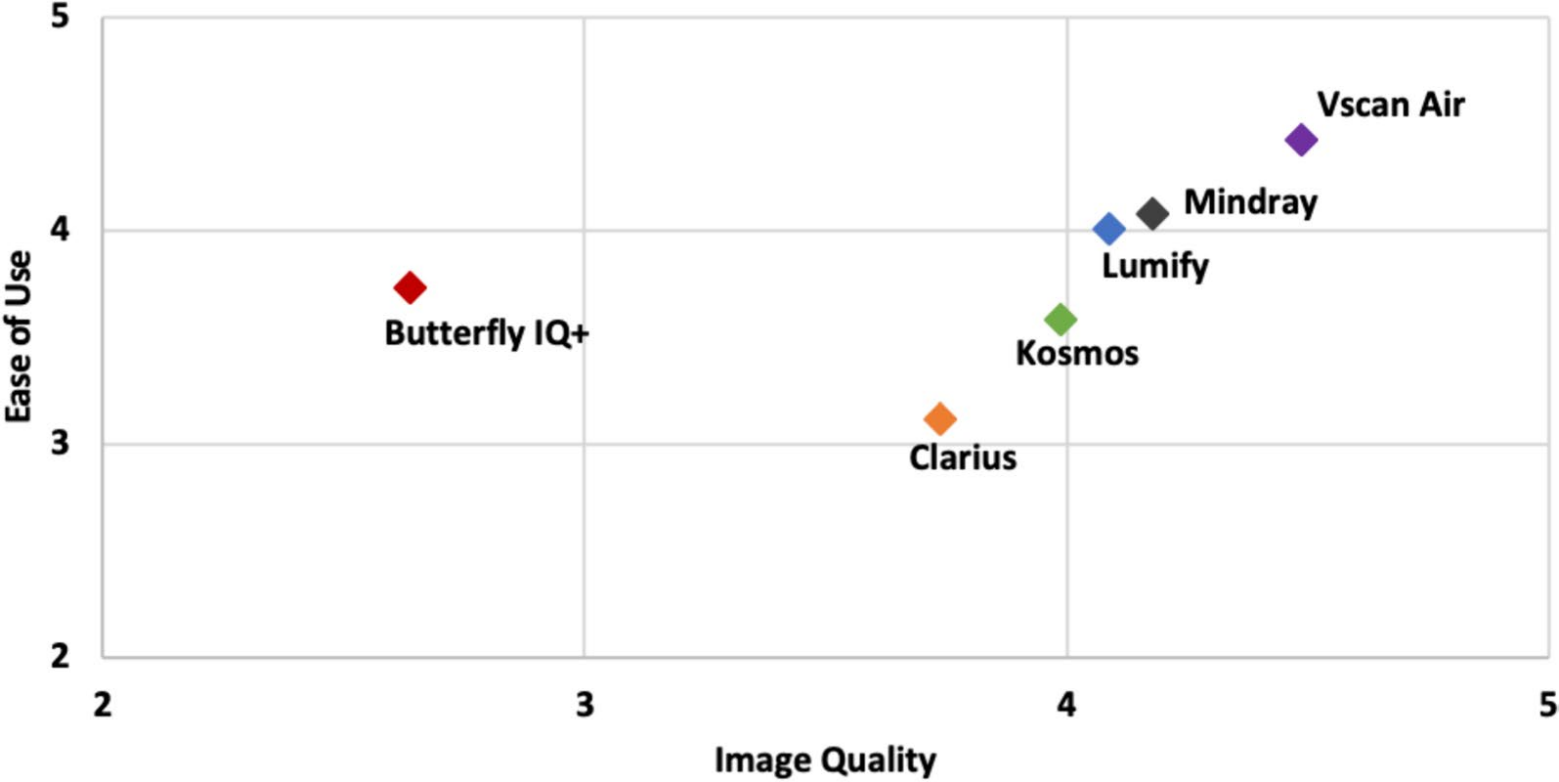
Mean Ratings of Handhelds by Ease of Use and Image Quality

For image quality, there were fewer statistically significant differences compared to ease of use. Vscan Air^™^ was rated highest in all categories (detail resolution, contrast resolution, penetration, clutter). For overall satisfaction with image quality, Vscan Air^™^, Lumify^™^, and Mindray were rated highest, and the differences were not statistically significant. A comparison of mean ratings for ease of use vs. image quality is illustrated in Fig. 5.

**Fig. 6:**
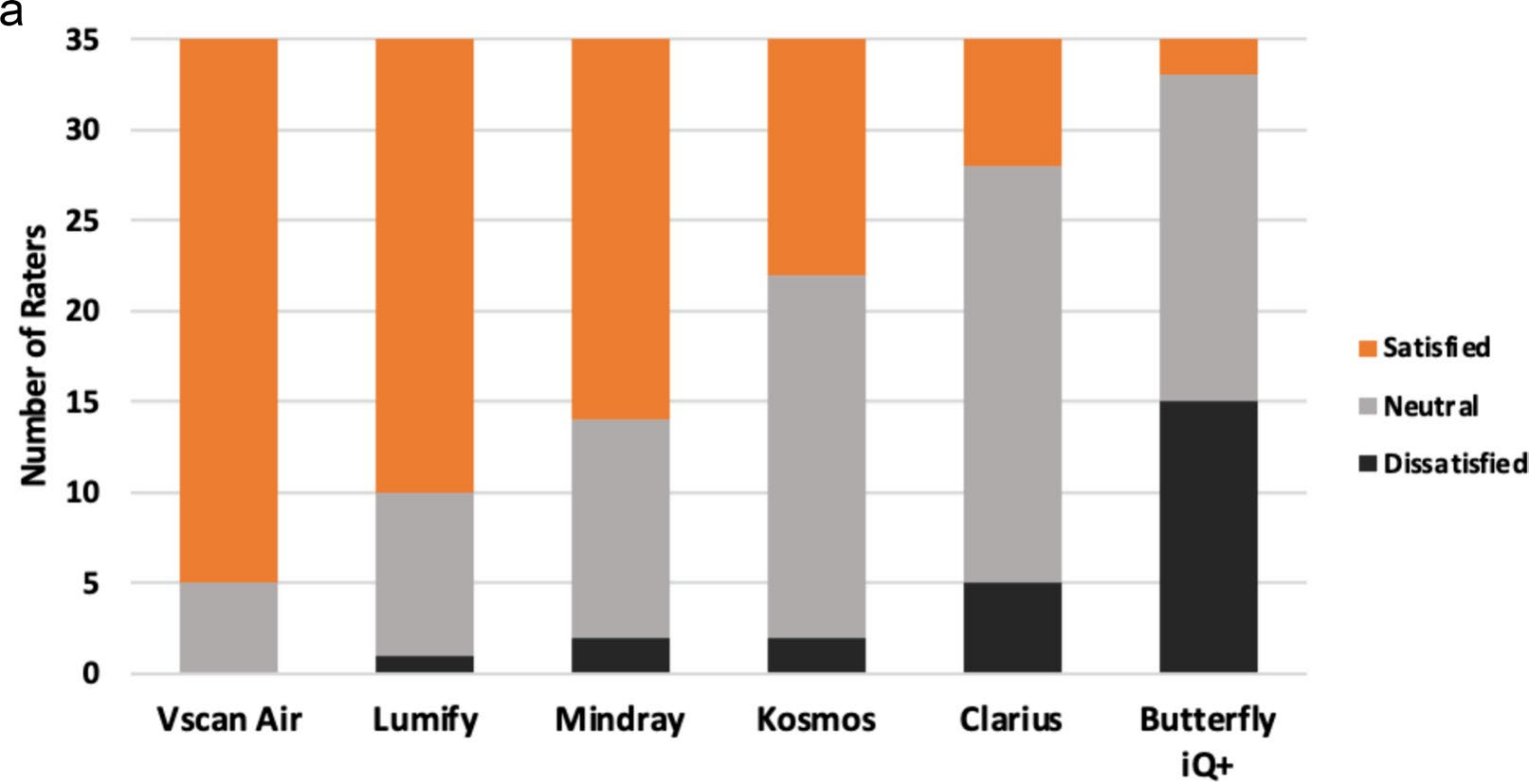

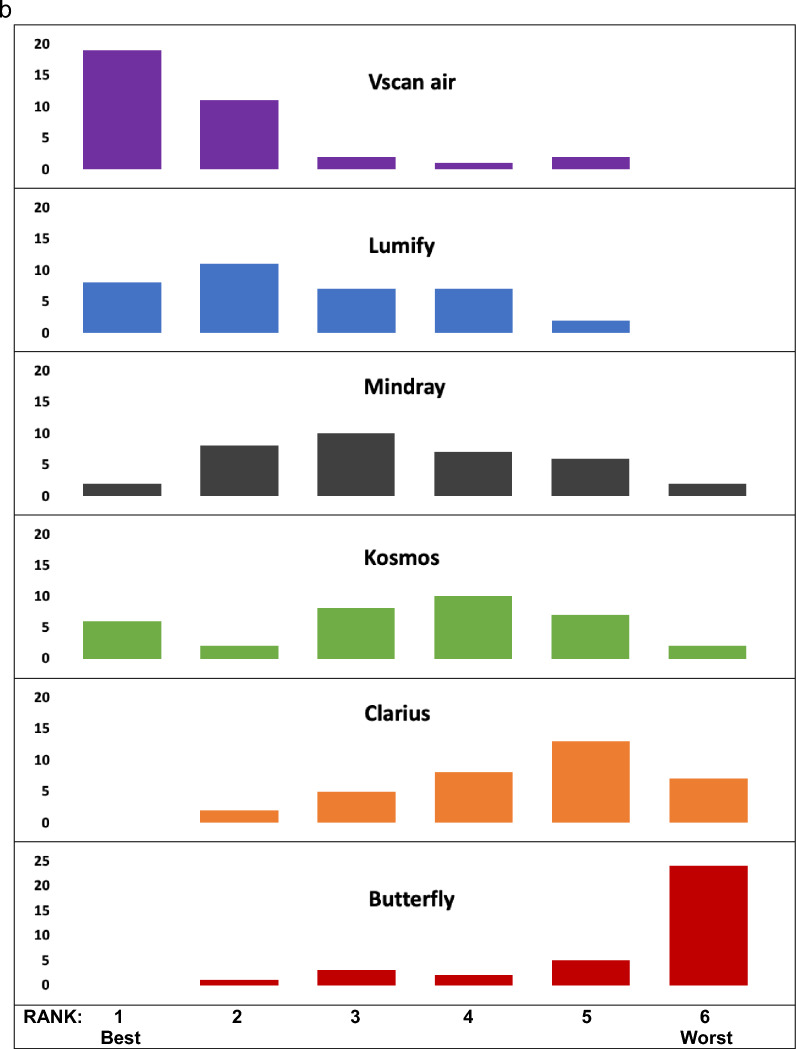

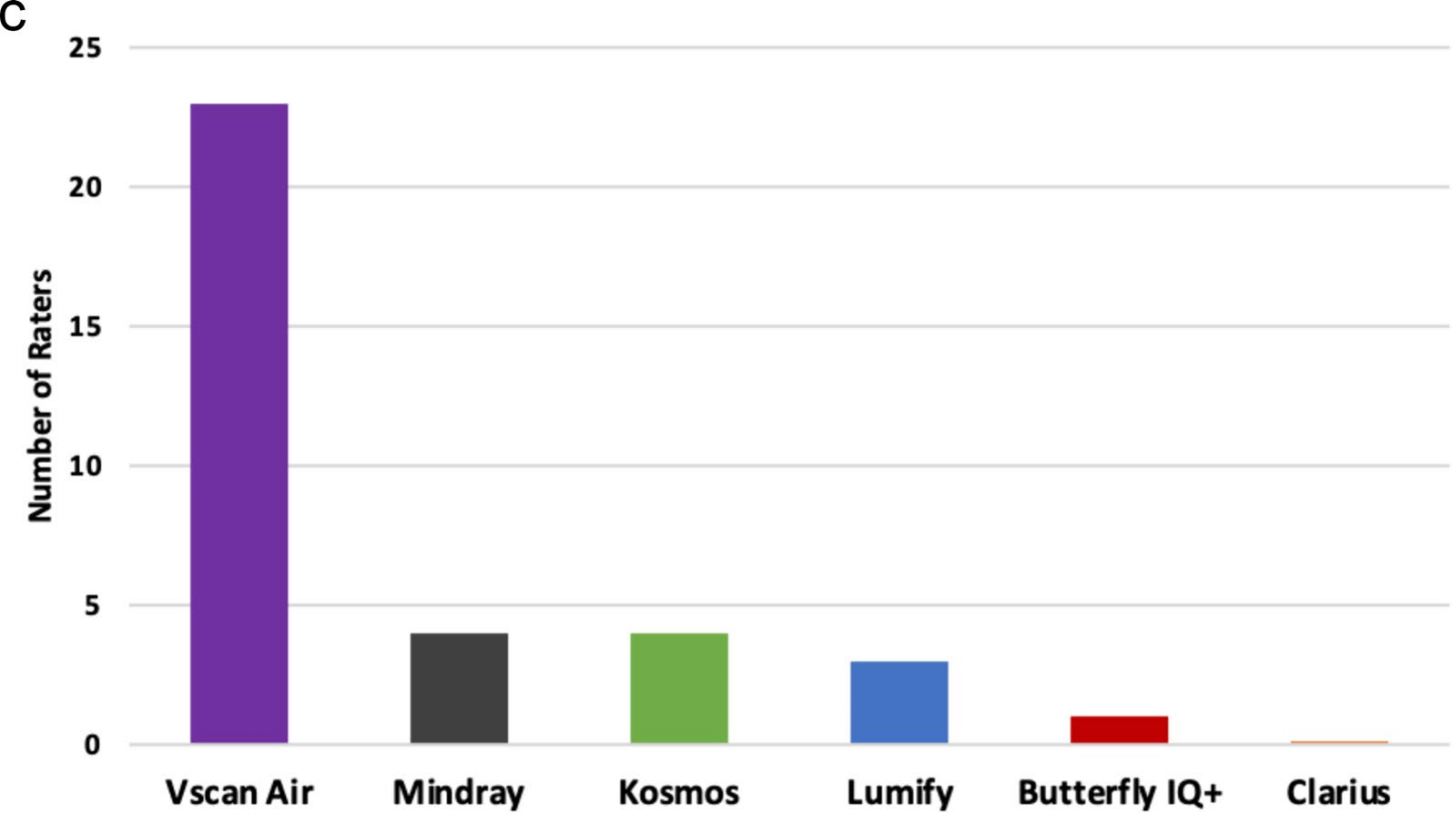
**A)** Overall Satisfaction with each Handheld Device; **B)** Overall Comparison Rankings of Handhelds by POCUS Experts; **C)** Purchasing Decision of Handheld to Carry in Pocket by POCUS Experts

The final survey asked experts about their overall satisfaction with each handheld (Fig. 6). Vscan Air^™^, Lumify^™^, and Mindray received the highest number of “satisfied” responses and ranked highest in order from 1 (“best”) to 6 (“worst”). When experts were asked which handheld they would purchase today as their personal device to carry in their coat pocket, a majority selected the Vscan Air^™^ (66%).

The 6 most important characteristics of handheld devices per experts were image quality, ease of use, portability, probe size, battery life, and availability of different probes. The least important characteristic was inclusion of artificial intelligence (AI) technology (Table 4).

**Table 4:** Importance of Characteristics of Handhelds per POCUS Experts

Characteristic	Very Important	Somewhat Important	Not Important
**Most important**			
1. Image Quality	35	0	0
2. Ease of Use	30	5	0
3. Portability	30	4	1
4. Probe Size	25	10	0
5. Battery Life^1^	22	13	0
5. Availability of Different Probes^1^	24	9	2
**Intermediate importance**			
6. Availability of Different Probes	24	9	2
7. M-mode, Color & Spectral Doppler	21	13	1
8. Total Costs^2^	20	14	1
8. Connectivity to Any Tablet or Phone^2^	21	12	2
9. Approved by Institution	21	8	6
10. PACS Integration	17	14	4
11. Software Calculation Packages	14	18	3
12. Customer Service (prior experience)	12	21	2
**Least important**			
13. Option for 1-time Purchase	14	16	5
14. Manufacturer’s Warranty	9	23	3
15. Wireless vs. Wired	12	16	7
16. Reputation of Manufacturer	11	14	10
17. Carrying Method (case vs. pocket)	7	21	7
18. Artificial Intelligence (AI) Technology	3	18	14

^1^ “Battery Life” and “Availability of Different Probes” were tied as the 5th most important characteristic

^2^ “Total Costs” and “Connectivity to Any Tablet or Phone” were tied as the 8th most important characteristic

Abbreviations: PACS, picture archiving and communication system; POCUS, point-of-care ultrasound

The qualitative data based on free-text comments from POCUS experts revealed a few important themes (Table 5). First, image quality is the most critical characteristic of handhelds because poor-quality images preclude making any clinical decisions. Thus, if an image of adequate quality to make a clinical decision cannot be obtained, it is not worth having the handheld. Second, after an adequate image quality can be acquired, it is desirable to have a small, multifunction (2- or 3-in-1), wireless probe. However, wireless probes that have connectivity issues, such as difficult, slow, or unreliable pairing with a tablet, are less desirable than wired probes. Finally, all 6 handhelds had notable advantages and disadvantages, and no single handheld was perceived as having all desired qualities or features.

**Table 5:** Advantages and Disadvantages of Handhelds per Comments of POCUS Experts (n = 35)

	Advantages (% respondents)	Disadvantages (% respondents)
Kosmos ^TM^	Good image quality (60%)	Large probe size (49%)
	Continuous and pulsed-wave Doppler (29%)	Wired (37%)
	Easy to use interface (23%)	Poor image quality (23%)
	AI functions (20%)	Difficult to use interface (17%)
		Multiple probes needed (14%)
		Cost (11%)
Vscan Air ^TM^	Good image quality (71%)	Connectivity issues (26%)
	Wireless connectivity (66%)	Poor image quality (20%)
	2-in-1 probe (60%)	Probe size/shape (17%)
	Easy to use interface (37%)	Limited spectral Doppler (no CWD) (14%)
Butterfly ^TM^	Easy to use interface (46%)	Poor image quality (89%)
	3-in-1 probe (34%)	Large probe size (60%)
	Cost (31%)	Membership fees (14%)
	Cloud storage (29%)	
Lumify ^TM^	Good image quality (77%)	Wired (54%)
	Small probe (40%)	Multiple probes needed (34%)
	Easy to use interface (34%)	Average Image quality (23%)
		Limited spectral Doppler (no CWD) (14%)
Mindray ^TM^	Good image quality (77%)	No linear probe (57%)
	Wireless (40%)	Difficult to use interface (43%)
	Probe size (37%)	Connectivity issues (14%)
		Probe size/shape (14%)
Clarius ^TM^	Good image quality (63%)	Large probe (74%)
	Wireless (40%)	Heat from probe (54%)
		Poor image quality (17%)
		Multiple probes needed (17%)

^*^Comments that were reported by < 10% (or ≤ 4) of POCUS experts were excluded

*CWD* continuous-wave Doppler, *POCUS* point-of-care ultrasound

### Bias evaluation

Potential bias due to prior experience with each handheld was assessed. Mindray and Clarius^™^ had a mean experience score < 1.1, indicating near total lack of experience with these devices. Kosmos^™^ and Vscan Air^™^ had mean experience scores of 1.5 and 1.6, respectively, indicating about half of experts had some experience. Lumify^™^ and Butterfly iQ + ^™^ had average experience scores of 2.1 and 2.4, respectively, indicating most users had some experience and several were proficient in their use.

No statistically significant association between experts’ experience levels and their ratings for image quality were seen (Additional File 6: Table S4). For ease of use, Vscan Air^™^ and Lumify^™^ had a small positive association with experience (correlation coefficient = 0.33, p = 0.05 for Vscan Air^™^ and correlation coefficient = 0.53, p = 0.001 for Lumify^™^), Thus, experts with more experience with Vscan Air^™^ and Lumify^™^ tended to rate them as being easier to use.

For overall satisfaction, there was no association with experience for five of the handhelds, but for Lumify^™^ there was a small positive association identified (correlation coefficient = 0.56, p = 0.001), indicating that experts with more experience tended to report more overall satisfaction with it. However, it is noteworthy that Butterfly iQ + ^™^ had the highest number of experts proficient in its use, yet it scored low in overall satisfaction. On the contrary, Mindray had virtually no experts with experience using it, yet it scored nearly equivalently as Lumify^™^ in overall satisfaction.

## Discussion

We compared the performance of 6 common handheld ultrasound devices for image quality, ease of use, and overall satisfaction. For image quality, the highest-rated handheld for the RUQ view was Vscan Air^™^, for the cardiac apical 4-chamber view was Mindray, and for superficial views of the neck and lung was Lumify^™^. The overall satisfaction with image quality was highest with Vscan Air^™^, Lumify^™^, and Mindray. The Vscan Air^™^ was rated highest for overall ease of use and was the most preferred handheld for purchase by POCUS experts. The most desirable characteristics of handhelds were image quality, ease of use, portability, probe size, battery life, and availability of different probe types.

Several studies have compared handhelds to cart-based ultrasound machines and have demonstrated similar accuracy for common diagnoses and procedures [18–31]. However, few studies have directly compared different brands of handhelds. [20, 32, 33] A study in 2020 compared 3 handhelds (GE Vscan^™^, Sonosite Iviz^™^, Philips Lumify^™^) for gynecologic measurements and common pathologies in patients and concluded that Lumify^™^ was the best handheld overall in this resource-limited setting [20]. Another study in 2024 evaluated 5 handhelds (Butterfly IQ +^™^, Clarius^™^ L15 and L20 probes, Lumify^™^, and Vscan Air^™^) with 3 ophthalmologists acquiring views of facial arteries, ocular/periocular structures, and areas for filler injections and concluded the Clarius^™^ L20 had the highest image quality for superficial facial structures [32]. Based on our review of the literature of handhelds, our group conducted the largest (n = 24) head-to-head comparison of handhelds for common general medical applications in December of 2021. [33] Building on our past work, the current study compared image quality based on specific characteristics of 3 common views, included new handhelds, and incorporated important hardware and software updates of existing handhelds. Further, by having a large number of POCUS experts (n = 35) conduct the handheld comparison on the same standardized patients, we were able to minimize potential patient, device, and operator variables that could confound results. Also, experts acquired and evaluated image quality in real-time as they would in clinical practice. Both high- and low-frequency transducers were used to assess abdominal, cardiac, and superficial views that are broadly relevant to clinical practice in multiple specialties.

**Fig. 7:**
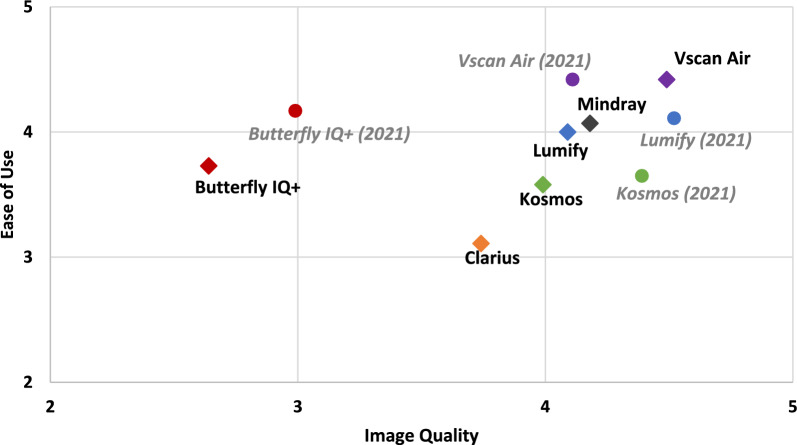
Comparison of Handheld Devices from December 2021 and January 2024. Mean ratings of ease of use and image quality are shown for Butterfly iQ +^™^, Kosmos^™^, Lumify^™^, and Vscan Air^™^ from 2021 and 2024. Mindray and Clarius^™^ were not included in the 2021 comparison study

Comparing data from our 2021 and present study revealed important similarities and differences. Most important, the distribution of data points in the graph comparing mean ease of use vs. image quality of handhelds has narrowed, signifying differences between handhelds appear to have become more subtle (Figs. 5 and 7). We anticipate the differences in image quality and ease of use between handhelds will continue to narrow and subsequently, other important characteristics, like battery life, probe ergonomics, and availability of different imaging modes, will differentiate the ratings of handhelds. Additionally, from 2021 to present, the Vscan Air^™^ surpassed Lumify^™^ with respect to overall satisfaction, and the Vscan Air^™^ continued to be the preferred handheld that experts would purchase “today as a personal device to carry in my coat pocket.”

Experts’ ratings of the most and least important characteristics of handhelds did not change significantly from 2021 to the current study. Among the 20 characteristics of handhelds, the 3 most important characteristics were image quality, ease of use, and portability, and the 5 least important characteristics only changed slightly in rank order. Although the most and least important characteristics of handhelds did not change significantly, no single handheld was perceived to have all desired characteristics, and all handhelds had important advantages and disadvantages (Table 5). For instance, wireless connectivity appeared to be preferred and advantageous, but when pairing between a handheld and tablet was slow or unreliable, wireless connectivity became a disadvantage. Furthermore, new AI functions have been added to most handhelds in recent years; however, experts rated AI technology as one of the least important characteristics of handhelds. Beyond handhelds, the current role of AI in medicine is unclear, and how clinicians will use AI in POCUS is yet to be determined. Perhaps AI will help facilitate self-directed POCUS training or allow less skilled clinicians to acquire and interpret POCUS images more accurately. For example, several handhelds and cart-based machines now perform automated cardiac calculations, and it is plausible that trainees or nurses could acquire cardiac measurements daily, similar to recording vital signs and other clinical parameters. Finally, adding new features to handhelds demands a critical balance of probe characteristics. If adding new features changes the probe size, weight, or costs substantially, the new feature may not be attractive to clinicians.

We acknowledge our study has limitations. First, we used standardized patients with a BMI < 24 and easily acquired views to minimize patient variables as confounders in the assessment of ease of use and image quality, but performance of these handhelds on patients with pathologic findings and higher BMIs may differ. For instance, we were unable to compare lung ultrasound performance using a low-frequency transducer to assess common lung pathologies, such as pneumonia and pulmonary edema. Second, bias from prior experience with some of the handhelds may have been a component in experts’ overall evaluation, but we did not identify a statistically significant correlation between experts’ prior experience and overall ratings of *image quality* of the devices. Bias from prior experience may have been a factor in the expert’s overall evaluation for *ease of use* of the devices, as the ratings for *ease of use* for Vscan Air^™^ and Lumify^™^ had a small positive association with experience. However, it is worth noting that Butterfly iQ + ^™^ had the highest number of experts proficient in its use, yet it scored low in overall satisfaction, and on the contrary, Mindray had virtually no experienced users, yet it scored nearly equivalently in satisfaction as Lumify. Third, the ultrasound manufacturers supplying handhelds for this study were requested to provide a tablet that best demonstrated their handheld device’s capabilities. However, handhelds were paired with tablets that varied in brand, operating system (iOS vs. Android), size, and resolution, and the selection of tablets may have affected experts’ ratings of image quality. Fourth, handheld purchasing decisions are complex, and this study focused on the 2 most important characteristics, image quality and ease of use. However, several device characteristics were rated as important, and though many of these characteristics appeared in our qualitative data, they were not addressed directly in our study, such as tablet connectivity and battery life from prolonged use. Notably, institutional approval of handhelds and integration with the local image archiving software or picture archival and communication system (PACS) may be the deciding factor for purchase of handhelds, regardless of the clinicians’ preferences.

## Conclusion

In our comparison of 6 handheld ultrasound devices, the overall satisfaction with image quality was rated highest with Vscan Air^™^, Lumify^™^, and Mindray. Specifically, image quality was rated highest with Vscan Air^™^ for the RUQ view, Mindray for the cardiac apical 4-chamber view, and Lumify^™^ for superficial views of the neck and lung. No single handheld ultrasound device was perceived to be superior in image quality for all 3 views. Vscan Air^™^ was rated highest for overall ease of use and was the most preferred handheld for purchase by POCUS experts. The most desirable characteristics of handhelds were image quality, ease of use, portability, probe size, battery life, and availability of different probe types. As differences in image quality and ease of use become less significant between handhelds, secondary characteristics, including portability, probe ergonomics, battery life, imaging modes, and costs, will become the distinguishing features of handhelds.

## Supplementary Information

Additional file 1. Abdominal Right Upper Quadrant View Data Collection Form

Additional file 2. Cardiac Apical 4-chamber View Data Collection Form

Additional file 3. Superficial Views of Neck & Lung Data Collection Form

Additional file 4. Overall Survey Comparing Handheld POCUS Devices

Additional file 5. Image Quality Ratings and Overall Ranking of Handhelds for Specific Views

Additional file 6. Individual Expert’s Experience with Devices Compared to Ratings for Overall Satisfaction, Image Quality, and Ease-of-Use

## Data Availability

The datasets used and/or analyzed during the current study are available from the corresponding author upon reasonable request.

## Abbreviations

CWD: Continuous wave Doppler
POCUS: Point-of-care ultrasound
PWD: Pulsed-wave Doppler
