# Supplementary material for: Comparison of 6 handheld ultrasound devices by point-of-care ultrasound experts: a cross-sectional study
Source: Ultrasound J. 2024 Oct 2;16:45. doi: 10.1186/s13089-024-00392-3 (PMC11447175; doi:10.1186/s13089-024-00392-3)
Supplement: Supplementary file 1 — Additional file 1. Abdominal Right Upper Quadrant View Data Collection Form [file 13089_2024_392_MOESM1_ESM.docx]

RATER NAME: _______________________________

MODEL Station Number: Handheld RUQ 1 Handheld RUQ 2 Handheld RUQ 3

|  | **POOR = 0**  Inadequate Quality | **INTERPRETABLE = 1**  Minimally Adequate | **GOOD = 2**  Adequate Quality | **EXCELLENT = 3**  Superior Quality |
| --- | --- | --- | --- | --- |
| Target Structures Visualized | Few | Some | All | All plus detail |
| Relative image quality | **Worse** than most handhelds | **Similar** to most handhelds | **Better** than most handhelds | **Similar to Cart-based** ultrasound machine |
| Ability to answer common clinical questions based on image | **NO!** No clinical questions could be answered with confidence | **Maybe** but some clinical questions could not be answered with confidence | **Yes,** most clinical questions could be answered with confidence | **YES!** All clinical questions could be answered with confidence |
| Could you make common clinical decisions based on this image? | “NO! I could **not make any clinical decisions** based on this image at all” | “Maybe – I could make **some clinical decisions** based on this image.” | “Yes, I could make **most clinical decisions** with confidence based on this image.” | “YES! I could make **all clinical decisions** with confidence based on this image.” |
| Need to obtain additional ultrasound imaging with cart-based machine | **YES!** I definitely would need additional US imaging with a cart-based machine | **YES**, I most likely would need additional US imaging with a cart-based machine | **NO**, I most likely would NOT need additional US imaging with a cart-based machine | **NO!** I definitely would NOT need additional US imaging with a cart-based machine |

Instructions:

1. Acquire a **right upper quadrant view** (liver, kidney, diaphragm) on model designated as “**Handheld: Abdomen**” with the **curvilinear OR phased-array probe** (if curvilinear not available) using the **abdominal exam** preset.
2. Use scale above (0-3) to **rate characteristics** of each handheld device in the table below.
3. For “Overall Ranking,” **rank the devices** from 1 (“Best”) to 6 (“Worst”) for this view.

|  | **Difference in echogenicity of renal cortex vs. liver** (0-3) | **Clarity of blood vessels in liver parenchyma** (0-3) | **Distinguish medullary pyramids in renal cortex** (0-3) | **Far-field resolution (spine, aorta)** (0-3) | **Color flow Doppler of vessels in renal pelvis** (0-3) | **OVERALL RANKING (1=Best; 6=Worst)** |
| --- | --- | --- | --- | --- | --- | --- |
| **Butterfly** |  |  |  |  |  | 1 2 3 4 5 6 |
| **Clarius** |  |  |  |  |  | 1 2 3 4 5 6 |
| **Kosmos** |  |  |  |  |  | 1 2 3 4 5 6 |
| **Lumify** |  |  |  |  |  | 1 2 3 4 5 6 |
| **Mindray** |  |  |  |  |  | 1 2 3 4 5 6 |
| **Vscan Air** |  |  |  |  |  | 1 2 3 4 5 6 |
| COMMENTS: |  |  |  |  |  |  |
