# Supplementary material for: Comparison of 6 handheld ultrasound devices by point-of-care ultrasound experts: a cross-sectional study
Source: Ultrasound J. 2024 Oct 2;16:45. doi: 10.1186/s13089-024-00392-3 (PMC11447175; doi:10.1186/s13089-024-00392-3)
Supplement: Supplementary file 5 — Additional file 5. Image Quality Ratings and Overall Ranking of Handhelds for Specific Views [file 13089_2024_392_MOESM5_ESM.docx]

**Additional File 5. Image Quality Ratings and Overall Ranking of Handhelds for Specific Views**

**Table S1. Abdominal Right Upper Quadrant View**

|  |  |  |  |  |  |  |
| --- | --- | --- | --- | --- | --- | --- |
| **View Characteristic Rated**^1^ | **Butterfly** | **Clarius** | **Kosmos** | **Lumify** | **Mindray** | **Vscan Air** |
| Difference in echogenicity of renal cortex vs liver | 1.86 | 2.11 | 2.11 | 2.31 | 2.14 | 2.63 |
| Clarity of blood vessels in liver parenchyma | 1.89 | 2.31 | 2.14 | 2.26 | 2.43 | 2.49 |
| Distinguish medullary pyramids in renal cortex | 1.77 | 1.86 | 1.89 | 2.14 | 2.06 | 2.29 |
| Far-field Resolution (spine, aorta) | 1.60 | 2.00 | 2.03 | 2.26 | 2.26 | 2.29 |
| Color Flow Doppler of vessels in renal pelvis | 1.69 | 2.09 | 1.83 | 2.23 | 2.14 | 2.37 |
| **Total Score^2^** | 8.80 | 10.37 | 10.00 | 11.20 | 11.03 | ***12.06*** |
| **Variability (s.d.)** | 0.12 | 0.17 | 0.14 | 0.06 | 0.14 | 0.15 |
| **Summary of Rankings** 1^st^  2^nd^  3^rd^  4^th^  5^th^  6^th^ | 2  2  3  6  2  20 | 5  7  7  5  6  5 | 6  2  3  9  14  1 | 7  9  6  7  3  3 | 5  6  10  4  6  4 | 11  11  6  4  3  0 |
| **Ranking Score^3^** | 76 | ***125*** | 114 | ***141*** | ***128*** | ***163*** |

^1^For each characteristic the user could rate the device as Poor (Inadequate)=0, Interpretable (Minimally Adequate)=1, Good (Adequate)=2, or Excellent (Superior)=3. The value presented is the average of 35 user ratings.

^2^Minimum possible total score is 0 and maximum possible total score is 15. Value presented is the total of the 5 means above it. The top score and any that are not significantly different from that score are designated in ***bold italic***.

^3^Minimum possible ranking score is 35 and maximum possible is 210. The top score and any that are not significantly different from that score are designated in ***bold italic***.

**Table S2. Cardiac Apical 4-chamber View**

|  |  |  |  |  |  |  |
| --- | --- | --- | --- | --- | --- | --- |
| **View Characteristic Rated**^1^ | **Butterfly** | **Clarius** | **Kosmos** | **Lumify** | **Mindray** | **Vscan Air** |
| Endocardial Definition | 0.94 | 1.60 | 1.89 | 2.03 | 2.29 | 2.11 |
| Clarity of Valve Leaflets | 1.20 | 1.71 | 1.94 | 2.23 | 2.46 | 2.29 |
| Clarity of Lateral TV Annulus | 1.06 | 1.46 | 1.91 | 2.17 | 2.26 | 2.23 |
| Far-field Resolution | 1.00 | 1.54 | 1.91 | 2.17 | 2.23 | 2.06 |
| Color Flow Doppler over LVOT or MV | 1.23 | 1.37 | 1.97 | 1.86 | 2.09 | 1.97 |
| **Total Score^2^** | 5.43 | 7.69 | 9.63 | 10.46 | ***11.31*** | 10.66 |
| **Variability (s.d.)** | 0.12 | 0.13 | 0.03 | 0.15 | 0.13 | 0.13 |
| **Summary of Rankings** 1^st^  2^nd^  3^rd^  4^th^  5^th^  6^th^ | 0  1  1  0  6  27 | 1  1  3  12  14  4 | 6  6  10  5  7  1 | 7  9  5  10  3  1 | 15  9  6  1  2  2 | 7  10  11  6  1  0 |
| **Ranking Score^3^** | 48 | 91 | ***136*** | ***144*** | ***168*** | ***156*** |

^1^For each characteristic the expert could rate the device as Poor (Inadequate)=0, Interpretable (Minimally Adequate)=1, Good (Adequate)=2, or Excellent (Superior)=3. The value presented is the average of 35 user ratings.

^2^Minimum possible total score is 0 and maximum possible total score is 15. Value presented is the total of the 5 means above it.

^3^Minimum possible ranking score is 35 and maximum possible is 210. The top score and any that are not significantly different from that score are designated in ***bold italic***.

**Table S3. Superficial Views of Neck & Lung Sliding^1^**

|  |  |  |  |  |  |
| --- | --- | --- | --- | --- | --- |
| **View Characteristic Rated**^2^ | **Butterfly** | **Clarius** | **Kosmos** | **Lumify** | **Vscan Air** |
| Clarity of carotid/internal jugular vein | 1.80 | 2.57 | 2.40 | 2.74 | 2.54 |
| Color flow Doppler of carotid/internal jugular vein | 1.97 | 2.11 | 1.97 | 2.26 | 2.46 |
| Difference in echogenicity of thyroid, vs vessels | 1.54 | 2.29 | 2.11 | 2.26 | 2.20 |
| Contrast of chest wall, vs pleural line | 1.51 | 2.17 | 1.94 | 2.51 | 2.29 |
| Clarity of lung sliding (“shimmering” of pleural line) | 1.69 | 2.14 | 1.89 | 2.54 | 2.26 |
| **Total Score^3^** | 8.51 | 11.29 | 10.31 | **12.31** | **11.74** |
| **Variability (s.d.)** | 0.19 | 0.19 | 0.21 | 0.21 | 0.14 |
| **Summary of Rankings** 1^st^  2^nd^  3^rd^  4^th^  5^th^ | 0  1  1  8  25 | 6  11  7  10  1 | 4  8  7  10  6 | 13  11  7  3  1 | 12  4  13  4  2 |
| **Ranking Score^4^** | 83 | ***151*** | ***134*** | ***172*** | ***160*** |

^1^The Mindray device has no linear probe and was not rated for this view.

^2^For each characteristic the expert could rate the device as Poor (Inadequate)=0, Interpretable (Minimally Adequate)=1, Good (Adequate)=2, or Excellent (Superior)=3. The value presented is the average of 35 user ratings.

^3^Minimum possible total score is 0 and maximum possible total score is 15. Value presented is the total of the 5 means above it.

^4^Minimum possible ranking score is 70 and maximum possible is 210. The top score and any that are not significantly different from that score are designated in ***bold italic***.
