# Supplementary material for: Comparison of 6 handheld ultrasound devices by point-of-care ultrasound experts: a cross-sectional study
Source: Ultrasound J. 2024 Oct 2;16:45. doi: 10.1186/s13089-024-00392-3 (PMC11447175; doi:10.1186/s13089-024-00392-3)
Supplement: Supplementary file 6 — Additional file 6. Individual Expert’s Experience with Devices Compared to Ratings for Overall Satisfaction, Image Quality, and Ease-of-Use [file 13089_2024_392_MOESM6_ESM.docx]

**Additional File 6.** Individual Expert’s Experience with Devices Compared to Ratings for Overall Satisfaction, Image Quality, and Ease-of-Use

|  | **Butterfly iQ+^TM^** | | | | | | **Clarius^TM^** | | | | | | | | | | | | **Kosmos^TM^** | | | | | | | | | |
| --- | --- | --- | --- | --- | --- | --- | --- | --- | --- | --- | --- | --- | --- | --- | --- | --- | --- | --- | --- | --- | --- | --- | --- | --- | --- | --- | --- | --- |
| **Expert** | *Exp* | | *Sat* | | *Ease* | *Qual* | *Exp* | | | | *Sat* | | | *Ease* | | | *Qual* | | *Exp* | | | *Sat* | | | *Ease* | | | *Qual* |
| **1** | 2 | | 1 | | 2.67 | 1 | 1 | | | 2 | | | 3.00 | | | 3 | | | 1 | | 2 | | | 3.33 | | 3.75 | | |
| **2** | 2 | | 2 | | 4.67 | 2.25 | 1 | | | 2 | | | 2.00 | | | 3 | | | 2 | | 2 | | | 4.00 | | 4 | | |
| **3** | 3 | | 1 | | 4.67 | 2.75 | 1 | | | 2 | | | 3.67 | | | 4 | | | 1 | | 3 | | | 4.00 | | 5 | | |
| **4** | 2 | | 1 | | 3.67 | 3.25 | 1 | | | 1 | | | 2.00 | | | 4 | | | 1 | | 1 | | | 2.67 | | 4.75 | | |
| **5** | 3 | | 2 | | 4.00 | 1.5 | 2 | | | 3 | | | 2.67 | | | 4 | | | 1 | | 2 | | | 3.00 | | 3.25 | | |
| **6** | 2 | | 1 | | 3.00 | 1.5 | 1 | | | 2 | | | 2.33 | | | 3 | | | 3 | | 3 | | | 3.67 | | 4 | | |
| **7** | 2 | | 2 | | 2.67 | 1.75 | 1 | | | 3 | | | 4.67 | | | 4.75 | | | 1 | | 3 | | | 4.00 | | 4.5 | | |
| **8** | 3 | | 1 | | 2.00 | 2 | 1 | | | 2 | | | 2.67 | | | 4 | | | 1 | | 2 | | | 3.67 | | 3.25 | | |
| **9** | 2 | | 2 | | 4.00 | 3.25 | 1 | | | 2 | | | 3.00 | | | 3 | | | 1 | | 2 | | | 3.00 | | 3 | | |
| **10** | 3 | | 1 | | 3.33 | 2 | 1 | | | 2 | | | 3.33 | | | 2.75 | | | 1 | | 2 | | | 2.67 | | 3 | | |
| **11** | 1 | | 2 | | 3.67 | 3.5 | 1 | | | 1 | | | 3.00 | | | 3.5 | | | 1 | | 2 | | | 3.67 | | 4 | | |
| **12** | 2 | | 2 | | 4.67 | 2.25 | 1 | | | 2 | | | 3.67 | | | 4 | | | 2 | | 2 | | | 4.67 | | 3 | | |
| **13** | 3 | | 2 | | 4.00 | 3.25 | 1 | | | 2 | | | 2.67 | | | 3.75 | | | 1 | | 3 | | | 4.67 | | 3.75 | | |
| **14** | 2 | | 1 | | 3.67 | 2.5 | 1 | | | 2 | | | 4.00 | | | 3 | | | 2 | | 2 | | | 4.33 | | 3.5 | | |
| **15** | 3 | | 2 | | 3.00 | 3 | 1 | | | 2 | | | 2.67 | | | 4 | | | 1 | | 3 | | | 3.33 | | 4.5 | | |
| **16** | 3 | | 2 | | 4.67 | 3.25 | 1 | | | 2 | | | 2.00 | | | 3.25 | | | 3 | | 2 | | | 4.00 | | 3.5 | | |
| **17** | 3 | | 1 | | 3.00 | 4 | 1 | | | 3 | | | 3.67 | | | 4 | | | 3 | | 3 | | | 4.00 | | 5 | | |
| **18** | 1 | | 1 | | 3.00 | 2.25 | 1 | | | 2 | | | 2.67 | | | 3.75 | | | 1 | | 2 | | | 3.00 | | 5 | | |
| **19** | 2 | | 1 | | 4.00 | 2.5 | 1 | | | 2 | | | 3.33 | | | 4 | | | 3 | | 3 | | | 4.33 | | 5 | | |
| **20** | 3 | | 1 | | 4.67 | 3.5 | 1 | | | 2 | | | 3.67 | | | 4 | | | 1 | | 2 | | | 4.00 | | 4 | | |
| **21** | 3 | | 3 | | 4.67 | 3.25 | 1 | | | 1 | | | 2.00 | | | 3 | | | 1 | | 3 | | | 5.00 | | 5 | | |
| **22** | 3 | | 2 | | 3.67 | 2.75 | 1 | | | 2 | | | 3.33 | | | 4 | | | 2 | | 2 | | | 2.33 | | 5 | | |
| **23** | 3 | | 2 | | 4.00 | 3.75 | 1 | | | 2 | | | 4.33 | | | 3.25 | | | 1 | | 2 | | | 3.67 | | 4 | | |
| **24** | 3 | | 2 | | 3.67 | 2.25 | 1 | | | 2 | | | 3.00 | | | 4 | | | 1 | | 3 | | | 1.67 | | 5 | | |
| **25** | 2 | | 1 | | 3.00 | 2.25 | 2 | | | 3 | | | 2.33 | | | 4.75 | | | 2 | | 3 | | | 2.67 | | 4.5 | | |
| **26** | 2 | | 2 | | 2.33 | 3.25 | 1 | | | 1 | | | 2.33 | | | 2.5 | | | 2 | | 2 | | | 3.67 | | 3.5 | | |
| **27** | 2 | | 2 | | 5.00 | 4.25 | 1 | | | 1 | | | 3.00 | | | 4 | | | 2 | | 2 | | | 3.33 | | 5 | | |
| **28** | 3 | | 2 | | 4.00 | 2.25 | 1 | | | 2 | | | 2.67 | | | 4.25 | | | 1 | | 2 | | | 4.00 | | 3 | | |
| **29** | 3 | | 2 | | 3.33 | 3 | 2 | | | 2 | | | 2.00 | | | 3 | | | 2 | | 2 | | | 3.67 | | 3.5 | | |
| **30** | 3 | | 2 | | 4.33 | 1.75 | 1 | | | 2 | | | 3.33 | | | 3.25 | | | 1 | | 2 | | | 3.67 | | 3 | | |
| **31** | 1 | | 1 | | 3.33 | 2.75 | 1 | | | 3 | | | 4.67 | | | 4.75 | | | 1 | | 2 | | | 3.33 | | 3.25 | | |
| **32** | 3 | | 3 | | 5.00 | 2.5 | 1 | | | 3 | | | 5.00 | | | 5 | | | 1 | | 3 | | | 4.00 | | 4.75 | | |
| **33** | 2 | | 1 | | 3.33 | 2 | 1 | | | 2 | | | 2.33 | | | 3.75 | | | 3 | | 3 | | | 4.00 | | 3.75 | | |
| **34** | 2 | | 2 | | 4.00 | 3 | 1 | | | 2 | | | 4.00 | | | 4 | | | 1 | | 1 | | | 2.33 | | 3 | | |
| **35** | 2 | | 1 | | 4.00 | 2.25 | 1 | | | 3 | | | 4.00 | | | 4.5 | | | 1 | | 3 | | | 4.00 | | 3.75 | | |
| **r_S_** |  | 0.31 | | 0.24 | | 0.09 | |  | 0.32 | | | -0.32 | | | 0.08 | | |  | | 0.16 | | | 0.25 | | | | 0.15 | |
| **p-val** |  | 0.067 | | 0.16 | | 0.61 | |  | 0.057 | | | 0.065 | | | 0.63 | | |  | | 0.37 | | | 0.15 | | | | 0.40 | |

|  | **Lumify^TM^** | | | | | | **Mindray^TM^** | | | | | | | | | | | | **Vscan Air^TM^** | | | | | | | | | |
| --- | --- | --- | --- | --- | --- | --- | --- | --- | --- | --- | --- | --- | --- | --- | --- | --- | --- | --- | --- | --- | --- | --- | --- | --- | --- | --- | --- | --- |
| **Expert** | *Exp* | | *Sat* | | *Ease* | *Qual* | *Exp* | | | | *Sat* | | | *Ease* | | | *Qual* | | *Exp* | | | *Sat* | | | *Ease* | | | *Qual* |
| **1** | 1 | | 2 | | 3.33 | 3.25 | 1 | | | 2 | | | 2.67 | | | 2.25 | | | 1 | | 2 | | | 3.33 | | 3 | | |
| **2** | 2 | | 3 | | 4.00 | 4 | 1 | | | 3 | | | 5.00 | | | 5 | | | 2 | | 3 | | | 5.00 | | 4 | | |
| **3** | 1 | | 3 | | 4.33 | 4.75 | 1 | | | 2 | | | 4.00 | | | 4 | | | 2 | | 3 | | | 4.67 | | 4.25 | | |
| **4** | 1 | | 2 | | 4.00 | 4 | 1 | | | 3 | | | 4.33 | | | 5 | | | 2 | | 3 | | | 5.00 | | 5 | | |
| **5** | 1 | | 3 | | 4.33 | 5 | 1 | | | 3 | | | 2.67 | | | 4.75 | | | 1 | | 3 | | | 3.67 | | 5 | | |
| **6** | 2 | | 3 | | 3.67 | 4 | 1 | | | 3 | | | 4.67 | | | 5 | | | 1 | | 3 | | | 4.33 | | 4.75 | | |
| **7** | 1 | | 2 | | 3.67 | 2.5 | 1 | | | 2 | | | 3.00 | | | 3 | | | 1 | | 3 | | | 4.33 | | 4.75 | | |
| **8** | 2 | | 3 | | 4.00 | 4 | 1 | | | 3 | | | 5.00 | | | 4 | | | 1 | | 2 | | | 2.33 | | 3 | | |
| **9** | 1 | | 2 | | 4.00 | 4 | 1 | | | 2 | | | 3.33 | | | 4 | | | 1 | | 2 | | | 3.00 | | 4 | | |
| **10** | 2 | | 3 | | 3.67 | 3.5 | 1 | | | 2 | | | 3.67 | | | 3.75 | | | 2 | | 3 | | | 4.00 | | 4 | | |
| **11** | 1 | | 2 | | 3.33 | 4.75 | 1 | | | 3 | | | 4.67 | | | 4 | | | 1 | | 3 | | | 4.33 | | 5 | | |
| **12** | 3 | | 3 | | 5.00 | 5 | 1 | | | 3 | | | 5.00 | | | 4.75 | | | 2 | | 2 | | | 5.00 | | 4.75 | | |
| **13** | 3 | | 3 | | 5.00 | 4.5 | 1 | | | 3 | | | 5.00 | | | 5 | | | 2 | | 3 | | | 5.00 | | 5 | | |
| **14** | 2 | | 3 | | 4.00 | 4 | 1 | | | 2 | | | 3.00 | | | 4.25 | | | 2 | | 3 | | | 4.33 | | 4 | | |
| **15** | 2 | | 3 | | 3.00 | 4.25 | 1 | | | 3 | | | 4.00 | | | 4.25 | | | 1 | | 3 | | | 5.00 | | 4.5 | | |
| **16** | 2 | | 3 | | 3.67 | 4 | 1 | | | 1 | | | 3.00 | | | 2.25 | | | 1 | | 3 | | | 4.67 | | 4.5 | | |
| **17** | 3 | | 3 | | 4.00 | 4 | 2 | | | 3 | | | 4.33 | | | 4 | | | 3 | | 3 | | | 4.00 | | 5 | | |
| **18** | 3 | | 3 | | 4.00 | 5 | 1 | | | 3 | | | 4.33 | | | 4.75 | | | 1 | | 3 | | | 4.67 | | 5 | | |
| **19** | 3 | | 3 | | 4.67 | 4 | 1 | | | 3 | | | 4.00 | | | 4.75 | | | 1 | | 3 | | | 4.67 | | 4.75 | | |
| **20** | 3 | | 3 | | 4.67 | 4.75 | 1 | | | 2 | | | 4.00 | | | 4 | | | 1 | | 3 | | | 4.67 | | 4.75 | | |
| **21** | 1 | | 2 | | 3.67 | 3.75 | 1 | | | 3 | | | 5.00 | | | 5 | | | 1 | | 2 | | | 2.33 | | 4 | | |
| **22** | 3 | | 3 | | 4.00 | 3.75 | 1 | | | 3 | | | 3.33 | | | 4.75 | | | 2 | | 3 | | | 4.67 | | 5 | | |
| **23** | 3 | | 3 | | 4.67 | 5 | 1 | | | 2 | | | 4.00 | | | 3.5 | | | 1 | | 3 | | | 5.00 | | 5 | | |
| **24** | 2 | | 3 | | 2.33 | 4.5 | 1 | | | 3 | | | 4.67 | | | 4.75 | | | 2 | | 3 | | | 4.67 | | 4 | | |
| **25** | 3 | | 3 | | 4.33 | 4 | 1 | | | 3 | | | 4.67 | | | 4.75 | | | 2 | | 3 | | | 5.00 | | 4.25 | | |
| **26** | 2 | | 2 | | 4.00 | 3.25 | 1 | | | 2 | | | 3.67 | | | 2.75 | | | 2 | | 3 | | | 4.33 | | 4 | | |
| **27** | 2 | | 2 | | 4.00 | 3.5 | 1 | | | 2 | | | 3.67 | | | 4.5 | | | 2 | | 3 | | | 4.67 | | 5 | | |
| **28** | 3 | | 3 | | 4.67 | 3.5 | 1 | | | 3 | | | 4.67 | | | 4.75 | | | 1 | | 3 | | | 4.67 | | 5 | | |
| **29** | 3 | | 3 | | 4.00 | 3.5 | 2 | | | 3 | | | 4.00 | | | 3.75 | | | 3 | | 3 | | | 4.67 | | 4 | | |
| **30** | 3 | | 3 | | 4.00 | 5 | 1 | | | 3 | | | 4.67 | | | 4.75 | | | 3 | | 3 | | | 5.00 | | 4 | | |
| **31** | 3 | | 3 | | 5.00 | 5 | 1 | | | 2 | | | 4.33 | | | 4.5 | | | 1 | | 3 | | | 4.67 | | 5 | | |
| **32** | 2 | | 3 | | 3.67 | 4.5 | 1 | | | 3 | | | 4.67 | | | 5 | | | 2 | | 3 | | | 4.67 | | 4.5 | | |
| **33** | 2 | | 2 | | 3.33 | 3.25 | 1 | | | 2 | | | 4.00 | | | 4 | | | 3 | | 3 | | | 5.00 | | 4.5 | | |
| **34** | 1 | | 3 | | 4.00 | 5 | 1 | | | 1 | | | 3.00 | | | 3 | | | 1 | | 3 | | | 5.00 | | 5 | | |
| **35** | 2 | | 1 | | 4.00 | 2.25 | 1 | | | 3 | | | 4.33 | | | 3.75 | | | 1 | | 3 | | | 4.33 | | 4.75 | | |
| **r_S_** |  | 0.56 | | 0.53 | | 0.19 | |  | 0.20 | | | 0.00 | | | -0.19 | | |  | | 0.24 | | | 0.33 | | | | -0.20 | |
| **p-val** |  | 0.001 | | 0.001 | | 0.28 | |  | 0.25 | | | 1.00 | | | 0.29 | | |  | | 0.16 | | | 0.05 | | | | 0.25 | |

***Exp****: Experience: 3=Extensive (“I use this device on a regular basis”), 2=Some (“I’ve used this device occasionally”), 1=None (“I’ve never used this device before”).*

***Sat****: Overall Satisfaction: 3=Satisfied (“I would use it in patient care”), 2=Neutral (“I might use it in patient care”), 1=Dissatisfied (“I would not use”).*

***Qual****: the average of 4 categories of Image Quality, each ranked on a scale of 1 to 5, with 5 indicating the most satisfaction.*

***Ease****: the average of 3 categories of Ease-of-Use, each ranked on a scale of 1 to 5, with 5 indicating the most satisfaction.*

***r_S_***  *is the Spearman correlation coefficient, and* ***p-val*** *is the p-value of the test of association based on the Spearman correlation coefficient.*
